# TFClass: a classification of human transcription factors and their rodent orthologs

**DOI:** 10.1093/nar/gku1064

**Published:** 2014-10-31

**Authors:** Edgar Wingender, Torsten Schoeps, Martin Haubrock, Jürgen Dönitz

**Affiliations:** 1 Institute of Bioinformatics, University Medical Center Göttingen, Georg August University, D-37077 Göttingen, Germany; 2 geneXplain GmbH, D-38302 Wolfenbüttel, Germany; 3 Johann-Friedrich-Blumenbach Institute of Zoology and Anthropology, Georg August University, D-37077 Göttingen, Germany

## Abstract

TFClass aims at classifying eukaryotic transcription factors (TFs) according to their DNA-binding domains (DBDs). For this, a classification schema comprising four generic levels (superclass, class, family and subfamily) was defined that could accommodate all known DNA-binding human TFs. They were assigned to their (sub-)families as instances at two different levels, the corresponding TF genes and individual gene products (protein isoforms). In the present version, all mouse and rat orthologs have been linked to the human TFs, and the mouse orthologs have been arranged in an independent ontology. Many TFs were assigned with typical DNA-binding patterns and positional weight matrices derived from high-throughput in-vitro binding studies. Predicted TF binding sites from human gene upstream sequences are now also attached to each human TF whenever a PWM was available for this factor or one of his paralogs. TFClass is freely available at http://tfclass.bioinf.med.uni-goettingen.de/ through a web interface and for download in OBO format.

## INTRODUCTION

DNA-binding transcription factors (TFs) regulate transcription by binding to genomic sites in regions of regulatory impact. In a complex interaction with enzymes that modify chromatin structure, mostly by methylation and acetylation events, they support the formation of the transcription preinitiation complex and direct the RNA polymerase to the transcription start site (TSS). The key function of TFs is to read out regulatory sequence signals in the genome and help transmitting them into the process of gene activation.

TFs recognize short specific sequence elements in a relaxed manner, which is frequently represented by positional weight matrices (PWMs). The way how the DNA-binding domains (DBDs) of TFs interact with their target sequences depends highly on the specific structural features of these domains. Different DBDs seem to have developed their own DNA–protein recognition code, which renders a systematic classification of DBDs a necessary prerequisite for any systematic characterization and prediction of protein–DNA interactions.

Exceeding the scope of previous catalogs of TFs and DBDs (1–6), we have introduced TFClass as a classification of human TFs based on their DBDs (7), which was a new version of a much older scheme that became part of the TRANSFAC^®^ database (8,9). With the recent updates to be reported here, we have introduced a number of smaller revisions in the structure, added mouse and rat orthologs of the human TFs in the classification, and present an independent ontology of mouse TFs, so far confined to the orthologs of the human TFs. Moreover, the information about TFs targets was enhanced by linking PWMs from a systematic in vitro screen (10), and lists of target sites and genes predicted with the TRANSFAC^®^ matrix library (11,12).

## DATA SOURCES

Domain assignments, protein sequences and information about isoforms were taken from UniProt, last update done using release 2014_07 (13), and TRANSFAC® (BIOBASE, Germany), with the last update using release 2014.2 (11). 3D structures were obtained from the PDB database (14), generally used as entry point to retrieve the original publications. The linked PWMs were taken from Jolma *et al.* (10). Domain annotations from UniProt were manually validated and edited where necessary. Sequence comparisons were basically done as described previously (7). In most cases, we used the BLAST option provided by the Expasy server (15) and the Clustal Omega tool implemented on the same server to check the similarities of presumed orthologs (16). As reported previously, protein expression signatures were composed using data from Protein Atlas, with the original data sets linked (17), and associated with the genus entries. Newly added are links to the corresponding BioGPS entries (18).

## STRUCTURE OF THE CLASSIFICATION

The overall structure of the proposed TF classification has not been changed since the last release (7), and will therefore be described only briefly. We have defined four taxonomic levels representing abstract concepts (superclass, class, family, subfamily) plus two that compile ‘tangible’ objects (genus, species). Definitions and explanations for these levels have been discussed previously (7,8).

Based on this structure and inspired by the EC numbering system for enzymes (19), we have assigned six-digit numbers to each TF molecule. Because of the generic meaning of the four top levels, we tried to make them sufficiently robust to be applied to a wide range of eukaryotic organism, if not universally. For instance, some class numbers have already been reserved for TFs from other organisms (Class 2.4, zinc cluster factors, known from fungal regulators; class 5.2, E2-related factors, known from papilloma viruses). Therefore, there are no differences among the superclass, class, family or subfamily definitions between human and mouse orthologs, although they may be differently populated by genera. This may be due to an as yet incomplete census of mouse TFs (see below), occasionally leading to empty subfamilies. We also cannot exclude yet that new subfamilies will have to be defined when we aim at generating the complete catalog of rodent TFs.

The two lower levels of the classification represent physical entities. A level 5 (‘genus’) entity is the group of polypeptides, usually just called ‘proteins’, that are encoded by the same gene. The individual TF polypeptides are listed as level 6 entities (molecular ‘species’). The relation of genera to their respective (sub-)family is ‘instance_of’, as is the relation of any particular polypeptide to its genus. These polypeptides, called species in TFClass, correspond to the isoforms in UniProt and proteins in TRANSFAC, whereas the genera of TFClass correspond to protein entries in UniProt, and to isogroups (i.e. groups of isoforms) in TRANSFAC. In a simplifying view, the genus level could be considered to represent TF genes, which would require to introduce a specific relation ‘encoded_by’ between level 6 and 5 entities. In TFClass, molecular species have been listed only when there are alternative products (usually: splice variants) known for a certain TF gene, otherwise they have been omitted for sake of clarity.

## HUMAN-RODENT ORTHOLOGS

### Identification of mouse and rat orthologs

As a first step to expand TFClass to other organisms than human, we tried to identify the rodent orthologs (mouse and rat, so far) of each human TF genus in UniProt and TRANSFAC®. Orthologous TF genes were identified according to the naming (including synonyms) of reviewed UniProt entries and sample checking by re-aligning the corresponding amino acid sequences. In case of unreviewed entries, alignment was routinely done. Two proteins were accepted as orthologs if (a) their domain structure was identical, and (b) their sequences coincided also outside the DBD, with a minimum of ∼50% identical amino acid residues, neglecting low-complexity regions (slight adaptations may have been necessary in individual classes). In addition, ortholog relationships were confirmed with the TRANSFAC® database 2014.2, where orthologous TF genes and their products (isogroups) are listed together as orthogroups. In case of unclear relationships, the human protein sequence in question was BLASTed against the whole UniProtKB database (20).

Also, the existing ortholog assignments of TRANSFAC were found to be reliable, but sometimes incomplete. In each case of missing orthologs in TRANSFAC but presumed orthologs in UniProt, the corresponding sequences were aligned and a decision was done whether to accept or reject them as orthologs. So far, we have not systematically used other sources of ortholog relationships such as KEGG (21), eggNOG (22), or others, since most of these resources apply different criteria and thresholds for accepting orthology. At this point, we didn't wish to enter the research field of identifying ‘real’ orthologs in evolutionary sense (23), having at hand sufficient evidence of linking with each other those TFs that apparently are the most similar ones in different organisms and can be justified to be placed in the same position of our classification.

### Classification of orthologs

This way, we have identified so far 1147 mouse and 1105 rat orthologs to human TFs, of which were 1065 mouse and 362 rat entities documented in reviewed UniProt entries. Because of this relatively low proportion of reviewed rat entries (33%), we decided to construct the whole classification first of all for the mouse orthologs only. Mouse (or rat) specific TFs have not yet been systematically captured.

**Figure 1.**
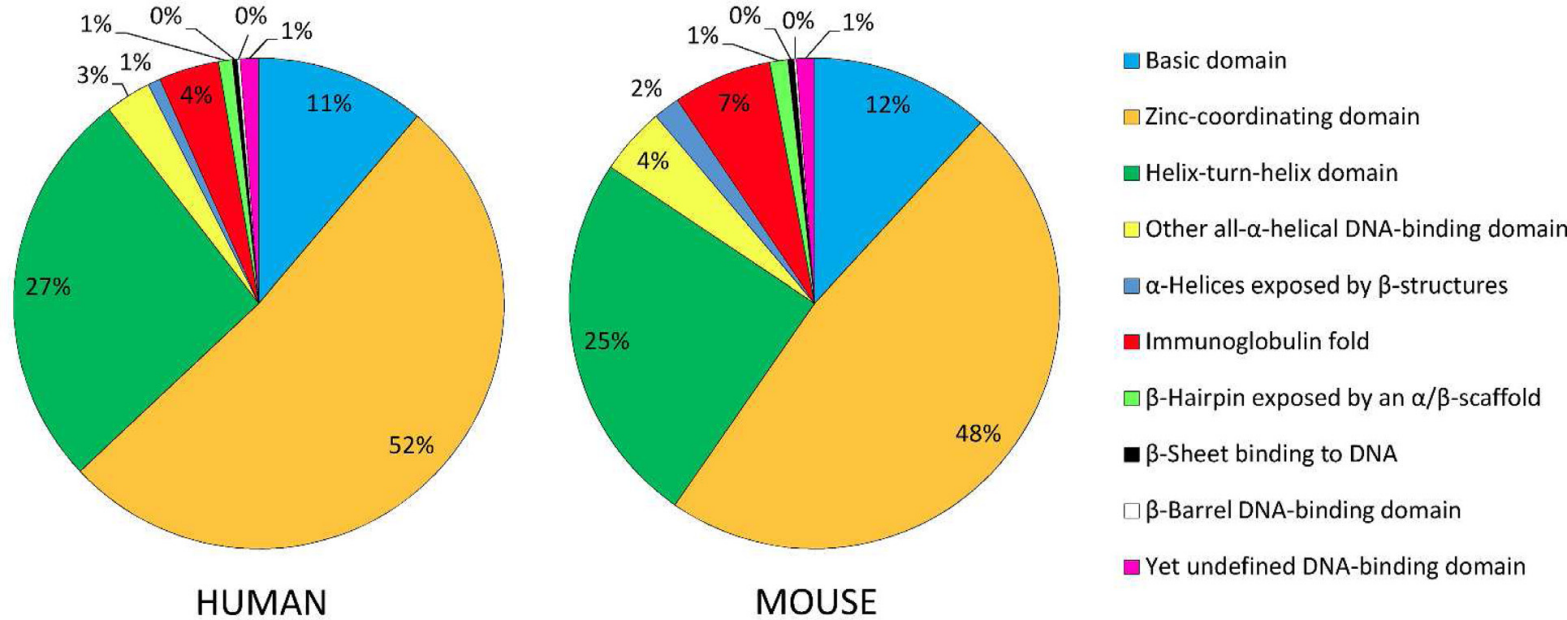
Superclass distribution of human TF genes and their mouse orthologs.

The classification scheme comprises 10 superclasses, which reflect the general topology of the DBD of the TFs and, thus, their interaction mode with specific DNA sequences (Table 1). Nine of them are defined, while the tenth (Superclass ‘0’) compiles TFs the 3D structure of which has not yet been solved, and which do not have substantial sequence similarity with other TFs, for which reason their assignment to any Superclass and Class, be it an existing or a new one, has not been possible so far. The three largest superclasses (1, 2 and 3) comprise 90% of all human or 86% of all orthologous mouse TF genera (Figure 1).

**Table 1.** The 10 superclasses of TFClass and their structure

Superclass No.	Superclass description	Classes	Families	Human				Mouse			
				Sub-families	Genera	Molecular species	Species/ genus	Sub-families	Genera	Molecular species	Species/ genus
**1**	Basic domain	3	18	36	174	387	2.22	36	174	259	1.49
2	Zinc-coordinating domain	8	25	130	807	1562	1.94	85	442	668	1.51
3	Helix-turn-helix domain	7	22	143	413	808	1.96	134	371	533	1.44
4	Other all-α-helical DBD	2	8	11	47	145	3.09	11	46	79	1.72
5	α-Helices exposed by β-structures	2	7	4	13	57	4.38	4	11	35	3.18
6	Immunoglobulin fold	7	16	6	62	213	3.44	6	62	130	2.10
7									
	β-Hairpin exposed by an α/β-scaffold	2	3	3	14	39	2.79	3	14	32	2.29
8	β-Sheet binding to DNA	2	2	0	5	13	2.60	0	5	6	1.20
9	β-Barrel DBD	1	1	3	3	5	1.67	3	3	5	1.67
0	Yet undefined DBD	5	10	0	19	39	2.05	0	19	34	1.79
	All	39	112	336	1557	3268	2.10	282	1147	1781	1.55

The table indicates the number of classes and families in each superclass as well as the number of human and orthologous mouse non-empty subfamilies, TF genera (TF genes) and molecular species (TF proteins). The last column in the human and mouse section gives the average number of isoforms per gene in each superclass.

For all of the previously defined 112 families, mouse orthologs were found, but there are a number of empty subfamilies in the classification of the mouse orthologs. Altogether, 54 out of 336 subfamilies that had been defined for the classification of human TFs did not exhibit any murine ortholog and also no correspondent factor in the rat genome.

While there is nearly a one-to-one correspondence of superclass 1 TF genera (173 identical genera in mouse and human with only 4 unreviewed mouse entries in UniProt; 1 bHLH TF genus specific for each human and mouse), a great discrepancy is to be observed in superclass 2 (Table 1). The number of TFs that is not conserved during evolution is particularly high among the C2H2 zinc finger proteins (Supplementary Table S1), as has been reported earlier even among primates (24). The five families of class 2.3 contain 45 subfamilies that are not occupied by any mouse ortholog, only 333 out of the 695 human C2H2 zinc finger proteins (genus level) have a matching ortholog in the mouse.

It should be stressed that identifying zinc finger human-rodent orthologs is subject to particular caveats. The first is about the naming convention: many human zinc finger proteins are named as ‘ZNF’ with a 1–3 digit number, while the denotation of most rodent zinc finger proteins is with ‘Zfp’ followed by a number. In many, if not most, cases, they correspond to each other. For instance, the sequence of human ZNF449 (UniProt entry Q6P9G9) is nearly identical with mouse and rat Zfp449 (Q8CB76 and M0R5T3, respectively; 462/518 positions = 89% identity, with the remaining positions subject to conservative exchanges). Usually, reviewed UniProt entries for the murine zinc fingers show both Znf and Zfp denotation, whereas the human entries have only the ZNF, and rat only the Zfp name. There are, however, clear exceptions: for instance, human ZNF781 (Q8N8C0) and mouse Zfp781 (Q0P5U5) are completely unrelated. Rat Zfp483 (Q99PJ8), although it has Znf483 as synonym, is unrelated to human ZNF483 (Q8TF39); the mouse ortholog has an unreviewed entry under the synonym Zkscan16. In other cases, the orthology between human ZNF and the similarly named mouse Zfp7 is at least questionable and has therefore been omitted, since either the number of zinc finger modules was different, or the sequence outside the anyway conserved zinc finger and accompanying motifs (such as KRAB domains) are highly divergent (e.g. human ZNF7 (P17097) and mouse Zfp7 (Q3TFZ4)). The evolution and concomitant classification of zinc finger proteins is an own research field (25,26,27) and will be subject of a separate review.

There are nine superclass 3 subfamilies that are empty in the classification of mouse orthologs. Most of them are sparsely occupied by a single human genus, whereas the DUX factors seem to be encoded by a complementary rather than an orthologous set of genes in the mouse (28), which will be included when we extend the classification of mouse orthologs to a comprehensive classification of all murine TFs.

In the other superclasses, all subfamilies found for human TFs are populated, and only for few genera no murine ortholog was found.

Because of the large diversity of the splice patterns of orthologous TF genes, the apparently much lower annotation density of mouse splice variants (1.55 i/o 2.10 species/genus) and the high dynamic of isoform annotation in UniProt, we decided to document the present state of known isoforms separately for both species. That implies that the sixth digit of the decimal classification numbers is specific for human and mouse polypeptides, equal numbers at that level do not indicate orthology. This may change in future when more robust data about the isoform patterns will be available for both human and mouse.

Altogether, the classification of human TFs (and the mouse orthologs, respectively) comprises now 1557 human (1147 mouse) genera and 3268 (1781) molecular species. The extent to which TF genes produce different gene products varies largely between the different superclasses and classes. In human, superclass 1 is only slightly above the total average species/genus ratio (Table 1), with a significant contribution of one bZIP family (CREB-related) and three bHLH families (E2A-related, PAS domain and bHLH-ZIP factors); all of them show much lower species/genus ratios in the mouse. Also superclass 2 as a whole is close to the overall average, but while the nuclear receptors are clearly above this value (3.31 species/genus), the large family of C2H2 factors is well below it (1.79). Especially, the nuclear receptors have nearly just half as many splice variants in the mouse than in human (1.73). Among the large superclasses, superclass 3 has the lowest species/genus ratio (1.96 in human, 1.44 in mouse), where the Hox-related factors are at the even lower end of the range (1.21 in human, 1.08 in mouse). The very high splice variability among the TF genes of some of the smaller superclasses is mainly due to the small number of genera, with some of them being subject to extensive alternative splicing (especially in superclass 5).

## DNA TARGETING BY TRANSCRIPTION FACTORS

### DNA-binding profiles

One of the most popular ways to represent the DNA-binding specificity of TFs are PWMs. There are several collections of such matrices such as TRANSFAC^®^ (11,29), JASPAR (30), HOCOMOCO (31) and more. Some of them have been generated by novel high-throughput screening approaches such the UniPROBE library (32). Recently, Taipale and co-workers published a large collection of matrices for human and mouse TFs generated with SELEX and ChIP-seq experiments (10). These matrices have been set in the TRANSFAC format and linked to the TFs of TFClass at genus level, along with additional information about the experimental details. A logo plot representation of one linked matrix is displayed first, which is then linked to the collection of matrices connected to a TF genus.

### Transcription factor binding sites

We combined promoter scanning with PWMs with a four-genome evolutionary conservation analysis to allocate presumed high-affinity, functionally significant TF binding sites (TFBS) in the 1-kb-upstream regions of all known human genes, referring to the TSSs annotated in RefSeq, and to infer TF-target gene relations (see (12) for details). Using all available vertebrate matrices of the TRANSFAC matrix library (release 2012.2), we predicted potential TFBSs by applying the Match program (33) with default minFN (‘minimize false negatives’) thresholds in order to retrieve the maximum of potential TF binding sites that have at least the quality of the TFBSs in the underlying training set of the corresponding matrix. Finally, we retrieved conserved binding sites for 1359 TRANSFAC defined matrices. These predictions were expanded to paralogous TFs to link also those members of the corresponding TF (sub-)family in TFClass for which no matrix is available yet. For each TF, or (sub-)family of TFs, where a matrix could be connected to, a list of these matrices can be called and for each of them, the list of predicted TFBSs can be displayed. These binding sites are given along with information about their chromosomal location, the associated gene, the TFBS sequence, the Match score and the strand orientation.

## IMPLEMENTATION

### Ontology backend and visualization

The classification is organized in three ontologies in the Open Biomedical Ontologies (OBO) format. One of them represents the concepts of the TF classification as ontological classes, independent of any concrete biological species and without any molecular equivalent. The two other ontologies represent the occurrence of specific TF genes and proteins in one organism, human (*Homo sapiens*) or mouse (*Mus musculus*). These two ontologies contain the fifth and sixth level of the classification and import the other levels from the first ontology.

To store the name and definition of the TFs the corresponding tags of the ontology, the xref tag is used for linking external resources. The rank of an item in the classification is mapped to six corresponding subsets.

To access the ontologies from the web application, the OBA (‘ontology-based answers’) service is used (34). This service maps the content of the ontologies to Java objects and provides pattern-based search using the name and IDs of the classification items. In addition, the subsets are used to expand the tree or part of it to a given level.

### TFClass interface

**Figure 2.**
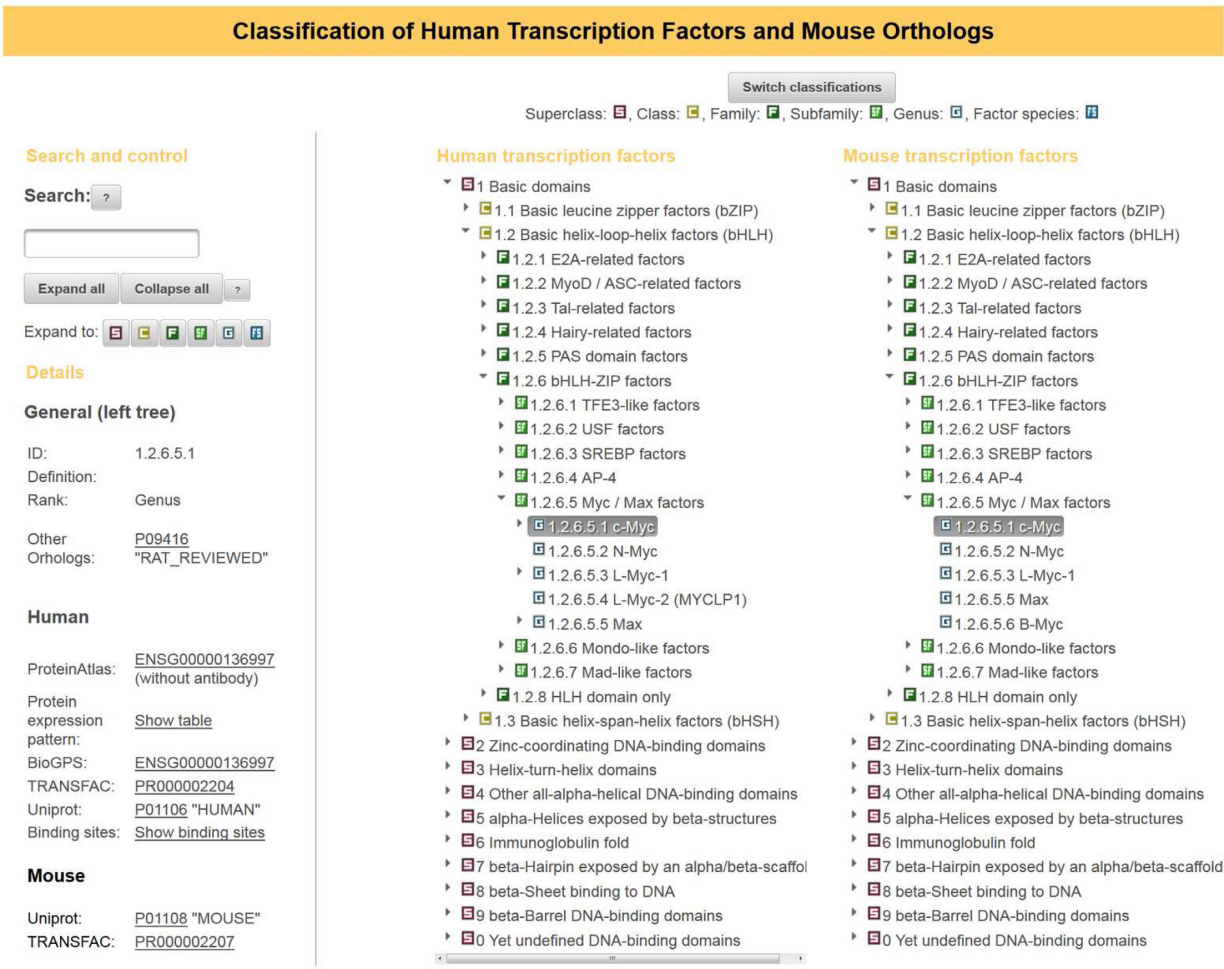
Web interface of human and mouse TFClass. In the center, the classification of human TFs is shown by default, on the right is the classification of mouse TFs. Navigating to a certain entity in the human classification (here: c-Myc) automatically opens the mouse classification to the same point. Note that the subfamily displayed here contains one human-specific factor (L-Myc-2, 1.2.6.5.4) in the central part and one mouse-specific TF (B-Myc, 1.2.6.5.6) on the right-hand side. On the left, additional information for the selected human TF is given, including external database links. The button ‘Switch classifications’ on top allows the user to put the mouse classification as primary one in the center, which would also switch the additional information on the left from human to mouse.

We have extended the previously described user interface (7) by an additional area exhibiting the mouse classification in addition to the human tree (Figure 2). The ontology in focus of the user (default: human) is displayed in the central part, the ontology of orthologs (presently: mouse) is shown on the right. Additional information is shown in the left part of the screen. It is specific for the level of the active entity, and some information is also specific for the organism in the central part of the display. When switching the main part to another scheme (at present only mouse available), the additional information on the left will change accordingly, while the human ontology will be shown on the right. New among this additional information are links to the PDB database (14).

Some of the additional information is too bulky to be displayed in the standard window. Instead, data about the expression profiles or the TF binding sites are opened in a separate overlay.

## AVAILABILITY

TFClass is freely accessible at http://tfclass.bioinf.med.uni-goettingen.de/ and has been made available in OBO format as a downloadable file. The latest drafts of the human and mouse classifications are also available as a fully expanded HTML document at http://www.edgar-wingender.de/huTFclassification.html and http://www.edgar-wingender.de/muTFclassification-1.html.

## SUPPLEMENTARY DATA

Supplementary Data are available at NAR Online.
